# Huntingtin and Its Role in Mechanisms of RNA-Mediated Toxicity

**DOI:** 10.3390/toxins13070487

**Published:** 2021-07-14

**Authors:** Annika Heinz, Deepti Kailash Nabariya, Sybille Krauss

**Affiliations:** Institute of Biology, University of Siegen, 57076 Siegen, North Rhine-Westphalia, Germany; Annika.Heinz@uni-siegen.de (A.H.); Deepti.Nabariya@uni-siegen.de (D.K.N.)

**Keywords:** huntingtin, RNA toxicity, CAG repeat, neurodegeneration, RNA hairpin, RNA binding protein (RBP), mis-splicing, translation, RNA-targeting compound

## Abstract

Huntington’s disease (HD) is caused by a CAG-repeat expansion mutation in the Huntingtin (HTT) gene. It is characterized by progressive psychiatric and neurological symptoms in combination with a progressive movement disorder. Despite the ubiquitous expression of HTT, pathological changes occur quite selectively in the central nervous system. Since the discovery of HD more than 150 years ago, a lot of research on molecular mechanisms contributing to neurotoxicity has remained the focal point. While traditionally, the protein encoded by the HTT gene remained the cynosure for researchers and was extensively reviewed elsewhere, several studies in the last few years clearly indicated the contribution of the mutant RNA transcript to cellular dysfunction as well. In this review, we outline recent studies on RNA-mediated molecular mechanisms that are linked to cellular dysfunction in HD models. These mechanisms include mis-splicing, aberrant translation, deregulation of the miRNA machinery, deregulated RNA transport and abnormal regulation of mitochondrial RNA. Furthermore, we summarize recent therapeutical approaches targeting the mutant HTT transcript. While currently available treatments are of a palliative nature only and do not halt the disease progression, recent clinical studies provide hope that these novel RNA-targeting strategies will lead to better therapeutic approaches.

## 1. Introduction

**Figure 1:**
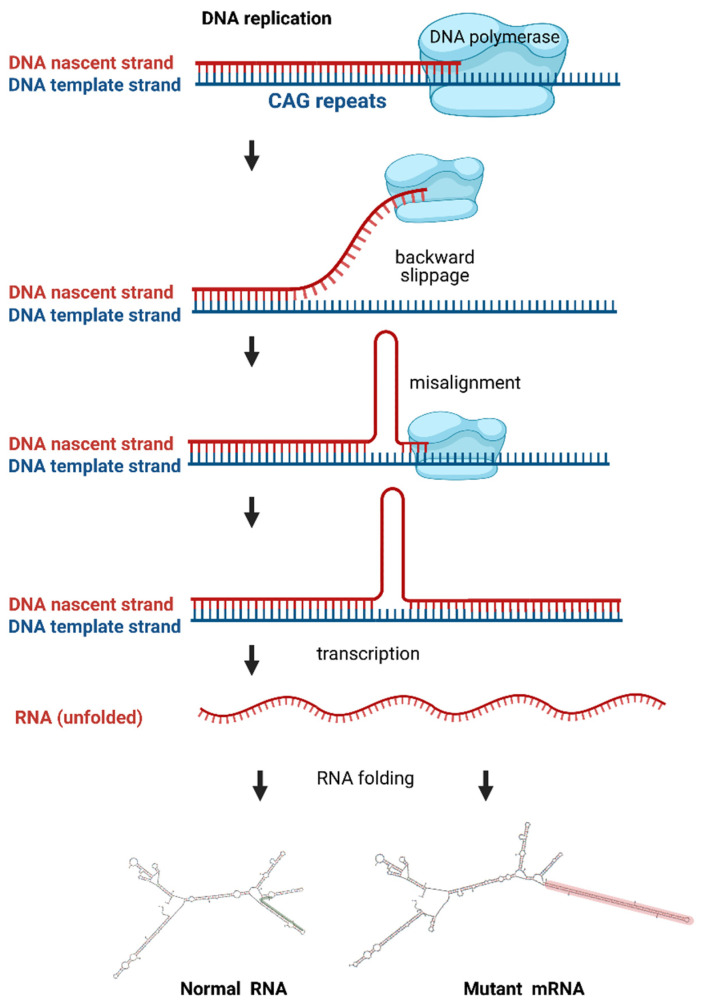
Schematic illustration of a CAG-repeat expansion during DNA replication, its transcription into RNA and three-dimensional RNA folding dependent on the CAG-repeat length. Upper part of the picture: During DNA replication, the polymerase uses the template strand (blue) to synthesize a nascent strand (red). CAG-repeat expansion during replication can be explained by strand-slippage. The polymerase detaches from the template strand and reattaches such that a part of the repeat sequence is looped out. Due to this misalignment, the nascent strand has an increased number of CAG repeats. Lower panel: the CAG repeat containing mRNA folds into a three-dimensional structure that depends on the repeat length. mRNA with either normal (left) or mutant (right) CAG repeat numbers are shown. The mutant CAG repeat folds into a characteristic hairpin structure that sticks out to the side. The mutant CAG-repeat hairpin is highlighted in red and the corresponding short CAG-repeat sequence in the normal transcript is highlighted in green. Created with BioRender.com and the RNA Folding Form V2.3 (http://www.unafold.org/mfold/applications/rna-folding-form-v2.php, accessed on 22 March 2021).

Short tandem repeats, such as trinucleotide-repeats, are a frequent motif in the human genome, with the trinucleotide CAG being one of the most common repeat motifs [1]. These repeats are polymorphic and variable in length. One proposed mechanism that is responsible for the variability is the so-called strand-slippage during DNA replication. This slippage is a consequence of a detachment of the DNA polymerase, followed by misalignment on the template strand with the repeated DNA fragment being looped out. If this mispairing between the template strand and the nascent strand occurs in such a way that the loop structure is built on the nascent strand, then the repeat number is elevated (Figure 1) [2].

Upon repeat expansion beyond a certain threshold, CAG-repeat expansion is associated with the development of neurodegenerative diseases [3,4]. CAG repeats, like all CXG repeats (with X being any nucleotide), are overrepresented in exons and occur most frequently within the open reading frame [5,6]. When translated into protein, CAG repeats code for polyglutamine tracts. Thus, CAG-repeat expansion disorders in which the CAG repeat is located in the coding region are also called polyglutamine diseases, with the most common one being Huntington’s disease (HD).

Clinical features of HD include three main groups: behavioral symptoms, cognitive difficulties and involuntary movements. The disease progresses over approximately 15–20 years and is ultimately lethal. Most patients (up to 50%) show some behavioral changes, such as depression, even before HD is diagnosed [7]. A high depression-associated suicide rate is seen in HD patients [8]. The rate of progression of early-onset psychiatric symptoms is variable and does not correlate with the rate of development of other symptoms, such as chorea and cognitive difficulties [9]. The cognitive difficulties often succeed initial behavioral changes, but at the time of diagnosis, most HD patients show significant cognitive impairment. This progresses slowly over many years, finally culminating in dementia. The third clinical feature in HD is chorea (from Greek χορεία, a circle dance). It describes a progressive movement disorder that is characterized by the loss of voluntary movements and the development of involuntary movements. The movement disorder starts with short suppressible unintended movements, for example, of the hands or the face. Later the involuntary movements affect more and more muscle groups as the disease progresses [10]. Other movement symptoms include bradykinesia and marked postural abnormalities [11].

In HD, the mutant CAG repeat is located within exon1 of the Huntingtin (HTT) gene on chromosome 4p16.3. The HTT gene product is a large protein of approximately 348 kDa. Upon expansion of the polyglutamine tract, the HTT protein, just like all polyglutamine proteins, tends to misfold and aggregate. HTT-polyglutamine protein aggregation in the central nervous system is a pathological hallmark of the disease and pathogenesis was consistently associated with the abnormal function of mutant HTT protein [12,13,14]. A well-established example of abnormal function of the mutant HTT protein is the down-regulation of the brain-derived neurotrophic factor (BDNF) via transcriptional alteration [15,16]. BDNF is involved in several processes in the nervous system, such as neuronal differentiation, synaptic plasticity, dendritic complexity and neuronal survival [17]; low levels of BNDF cause degeneration of striatal projection neurons in HD [16,18,19]. Because proteolytic cleavage of mutant HTT protein is an established rate-limiting step in the aggregation process, a lot of research has focused on the N-terminal fragment of the mutant HTT protein that contains the polyglutamine tract. Interestingly, the second cleavage product of mutant HTT, the non-polyglutamine C-terminal fragment, was also reported to cause cellular toxicity [20]. However, the role of aggregates and aggregate-prone polyglutamine protein in pathogenesis was reviewed extensively elsewhere [21,22,23,24,25] and we will focus on RNA-mediated molecular mechanisms that are linked to cellular dysfunction in HD in this review.

In the last couple of years, there has been emerging evidence indicating the contribution of RNA-mediated abnormal functions to pathogenesis. Several lines of evidence indicate that mutant CAG repeats can induce RNA-mediated toxicity in in vivo models expressing untranslated repeats [26,27,28,29,30]. In addition, there are also examples of human diseases, in which an expanded CAG repeat is located in the untranslated region, suggesting that CAG-repeat RNA can cause disease development, even in the absence of a polyglutamine protein (these diseases include SCA12 (spinocerebellar Ataxia Type 12, OMIM 604326), SCA8 (spinocerebellar ataxia type 8, OMIM 608768) and Huntington disease-like 2 (OMIM 606438)).

## 2. RNA Structure and Misfolding

**Figure 2:**
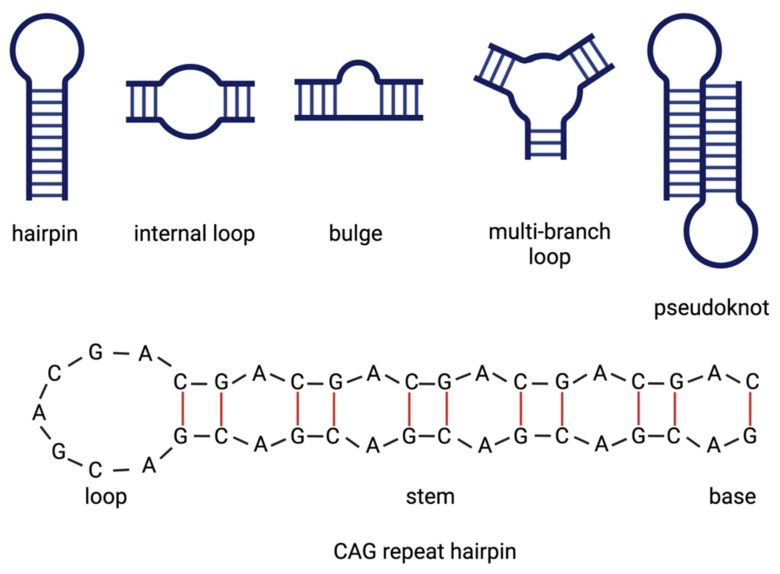
Schematic illustration of three-dimensional RNA folding structures. RNA can form fully paired and non-canonically paired regions, such as hairpins, internal loops, bulges, multi-branch loops and pseudoknots (**upper panel**). CAG-repeat regions can fold into hairpins in which the opposing strands of the CAG-repeat form Watson–Crick contacts between the G and C positions (indicated as red lines) and wobble pairs at the A–A mismatches (**lower panel**). In this picture, a terminal loop of seven nucleotides is shown. Created with BioRender.com.

One way of explaining a toxic gain-of-function of an RNA molecule is by aberrant attachment to proteins or other RNAs, where the three-dimensional structure is the key for intermolecular binding. RNA is most often a single-stranded molecule that folds onto itself to minimize its free energy. It can form fully paired and non-canonically paired regions, such as hairpins, internal loops, bulges, multi-branch loops and pseudoknots (Figure 2). These structural motifs are important recognition sites for RNA–protein and RNA–RNA interactions. While the three-dimensional structure of an RNA molecule is crucial to its physiological function, its misfolding can lead to the deregulation of various cellular processes, resulting in a toxic gain-of-function under disease conditions [31]. With respect to CAG-repeat RNA, a toxic gain-of-function is triggered by an aberrantly folded RNA hairpin motif in the repeat location that is not present under healthy conditions. Hydrogen bonding between nucleobases plays a crucial role in RNA folding. In the expanded CAG-repeat regions, the RNA folds onto itself with the opposing strands of the CAG repeat, forming classic Watson–Crick contacts between the G and C positions and wobble pairs at the A–A mismatches (Figure 2). The stability and the length of the hairpin are dependent on the number of CAG repeats. While in non-mutant HTT transcripts, the CAG repeat pairs with an adjacent CCG repeat, expansion of the CAG repeat leads to the folding of a double-stranded hairpin structure of pure CAG repeats. The hairpin can be divided into three parts: the base of the hairpin, the stem region that is built up of the double-stranded CAG region and a terminal loop of either four or seven nucleotides. The size of the terminal loop depends on the repeat number, with even numbers mainly resulting in loops of four nucleotides and odd numbers mainly resulting in loops of seven nucleotides. Not only do the CAG-repeat lengths influence hairpin stability but also specific repeat-flanking regions at the stem can stabilize the hairpin structure by serving as a G–C clamp [32]. These aberrantly formed mutant CAG-repeat hairpin motifs interfere with the normal cellular functions by aberrantly recruiting RNA-binding proteins [32,33]. The sequestration of these proteins to the hairpin hinders the function of several pathways they are a part of, leading to the breakdown of the cellular system.

## 3. RNA Localization and RNA Granules

An essential part of spatial protein translation control is the regulation of cytoplasmic mRNA localization. RNA is transcribed in the nucleus and, after maturation into mRNA, binds to specific proteins that trigger mRNA export to the cytoplasm, where the mRNA can be translated into protein. The discovery that mutant CAG repeats promote nuclear retention and formation of RNA foci brought a new vision in the field of neurological disorders. Apparently, the mutant CAG-repeat RNA gets retained inside the nucleus due to aberrant RNA–protein interactions. Such interactions may pave the way to strong sequestration of other proteins, such as muscleblind 1 (MBNL1), which is an important protein in foci formation [34]. RNAs with CAG-repeat expansions were found within foci, along with MBNL1 in HD cells [35]. RNA foci occur in various cell models of HD, such as lymphoblasts, fibroblasts and neuronal progenitors [36]. The presence of RNA foci leads to alterations in cellular pathways, apoptosis initiation and abnormal alternative splicing [37]. However, whether the nuclear accumulation of CAG-repeat transcripts represents a stable fraction that remains in the nucleus or whether CAG-repeat transcripts travel to the cytoplasm after temporary participation in the formation of foci remains unclear [35].

One important phenomenon that leads to RNA toxicity upon CAG-repeat expansion and that is linked to aberrant RNA localization is RNA granulation. The maintenance of neuronal circuits and synaptic strength is largely dependent on the local translation of mRNAs [38]. Neuronal RNA granules are a transport mechanism of mRNAs and other proteins to the synapses. Pertaining to their complex morphology, neurons rely on various internal factors to achieve precise compartmentalization of external signals. This is partially achieved by decentralizing the control of gene expression and by the dynamic local translation of mRNA that is transported to axon terminals and dendrites. Both the transport and the translation of these mRNAs are tightly regulated. During transport, mRNA translation is repressed inside the transport cargo. The release of translational repression is temporally and spatially controlled and triggered either by signaling molecules or by synaptic activity. Although the exact mechanistic process remains an integral part of several ongoing studies, it is clear that neuronal RNA binding proteins are required for the targeting of mRNA and translational regulation. They recognize the regulatory sequences, recruit translational repressors and enable the formation of multi-molecular ribonucleoprotein complexes (RNPs). These RNPs assemble to form neuronal RNP granules. In addition to these RNP granules, neuronal cells also contain other cytoplasmic RNP granules that include processing bodies (PBs) and stress granules (SGs). However, these different classes of granules sometimes contain common proteins in their cargo and use similar mechanisms to control mRNA translation or decay [39,40]. Neuronal granules often contain large amounts of ribosomal and other RNA-binding proteins. Proteins such as FMRP (fragile X mental retardation protein), Staufen and UPF1 are key regulators of translational silencing and activation in neurons [40]. Often, these proteins are trapped by the mutant HTT mRNA, leading to the breakdown of the transport machinery. Studies by Savas et al. suggest that the HTT protein also plays an important role in RNA transport and translation within neuronal granules [41]. The mutant RNA obstructs the axonal transport and compromises the synaptic excitability of the neuron. This failure of receptor delivery may lead to excitotoxic damage of the neuron [42].

PBs are formed via phase separation within the cytoplasm and play a role in mRNA degradation [43]. They also serve as sites for miRNA-mediated translational silencing [44,45]. Ago2 is an integral protein that facilitates neuronal HTT-mediated gene silencing and copurifies with the HTT protein. HTT and Ago2 associate and localize themselves in PBs in order to contribute to post-translational gene silencing [41]. Endogenous HTT protein also associates with Ago2 in neuronal granules and supports the transport of mRNA to dendrites. The knockdown of HTT results in improper Ago2 distribution. Previous studies also reported that the integrity of PBs is compromised in striatal cells and primary neurons that express endogenous mutant HTT [41].

SGs are dense attachments of RNAs and RNA-binding proteins that form in response to cell stress. They can be seen as a storage granule for untranslated mRNAs. The stored mRNAs can go down one of three paths to promote cell survival: further storage, re-initiation of translation or decay [46]. To facilitate degradation, SGs transfer transcripts to the adjacent PBs [47]. The pathophysiology of most neurodegenerative disorders includes the presence of oxidative stress. In response to oxidative stress, eukaryotes either activate defense mechanisms that help cell survival or initiate apoptotic pathways [48]. SGs are nucleated through the binding of core mRNA-binding proteins (RBPs) to mRNA. These core RBPs often contain polyglycine-rich domains and can recruit proteins that are linked to several neurodegenerative disorders [49]. RBPs include several molecules, such as optineurin, angiogenin, ataxin-2, hnRNPA1, SMN-1, TIA-1, TDP-43, TTP, FUS and B2, which are known to associate with SGs. Mutations and malfunctions in these RBPs could cause direct neurodegeneration [50,51].

## 4. Deregulated Splicing

One approach to identifying mechanisms of RNA-mediated toxicity of mutant CAG-repeat transcripts is to analyze the proteome that aberrantly binds to expanded CAG repeats. Schilling et al. purified proteins that are captured by HTT RNA in a repeat-length-dependent manner and identified them using mass spectrometry [52]. Of the 36 proteins binding HTT RNA with a higher affinity for RNAs harboring mutant CAG repeats, 32 proteins are functionally connected to splicing. This finding indicates that one major mechanism of RNA-mediated toxicity is deregulated splicing. Indeed, several mis-splicing events were detected in different models of HD.

**Figure 3:**
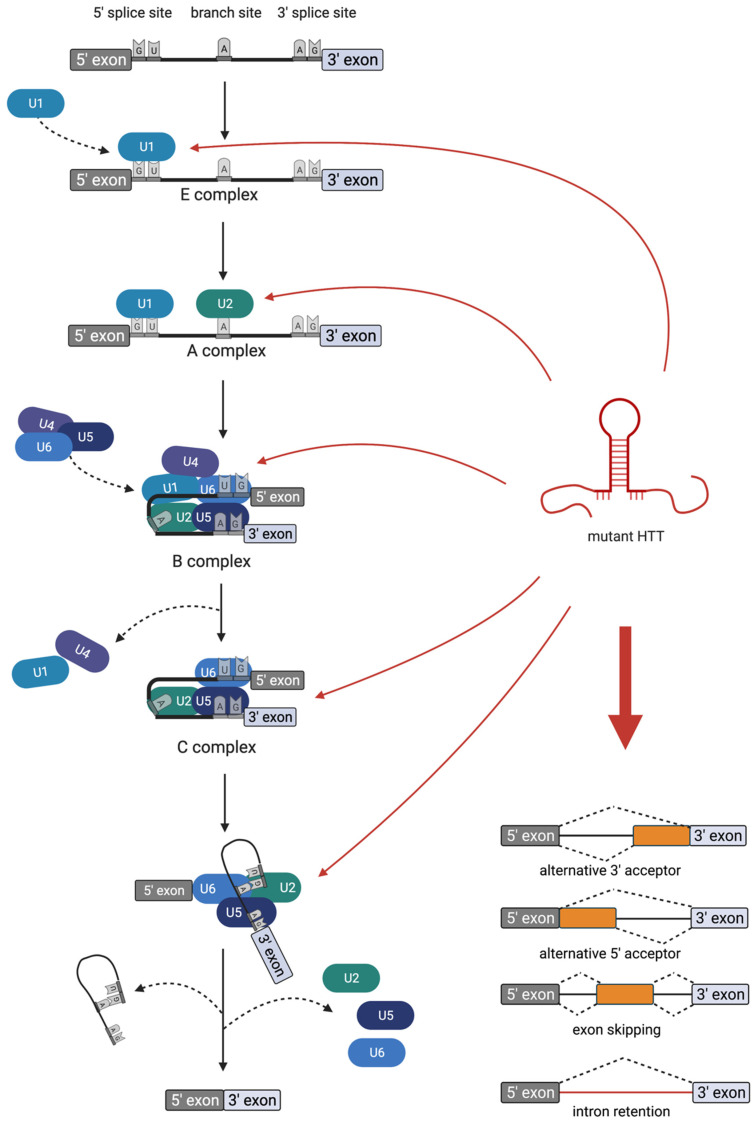
Proteins that aberrantly bind to HTT RNA with expanded CAG repeats regulate splicing. A schematic of the splicing process is shown. RNA splicing is a process that removes non-coding sequences (introns) from pre-mRNA and joins the protein-coding sequences (exons) together, which is carried out by the spliceosome. The spliceosome consists of several proteins and small nuclear RNAs. The major spliceosome includes the small nuclear ribonucleoproteins (snRNPs) U1 (blue), U2 (green), U4 (violet), U5 (dark blue) and U6 (blue). These snRNPs sequentially attach to the pre-mRNA and carry out splicing, starting with U1, which binds at the 5′ splice site of the exon. U2 assembles at the branch site and then the U5 and U4–U6 complexes attach, resulting in the for-mation of the precatalytic spliceosome. Afterward, U4 and U1 are released from the pre-spliceosomal complex, the 5′ splice site gets cleaved and a looped lariat structure containing the intronic sequence is removed. Finally, the two exons are linked. HTT RNA with an expanded CAG repeat (hairpin depicted in red) aberrantly sticks to the snRNPs U1, U2, U4, U5 and U6. This results in aberrant splicing events, including intron retention, exon skipping or the use of alternative 3′ or 5′ acceptor sites. Created with BioRender.com (accessed on 22 March 2021).

Aberrant splicing events that occur upon the expression of mutant CAG-repeat RNA can be generally grouped into two groups: in splicing events affecting the CAG-repeat transcript itself and in splicing events affecting other transcripts. Both groups of mis-splicing events were shown in different CAG disease models (Figure 3).

The first group, namely, mis-splicing of the mutant CAG-repeat transcript itself, occurs in diverse HD models and disease tissues. Here, the partial splicing of mutant HTT from exon 1 to exon 2 leads to the production of a small polyadenylated transcript encoding a neurotoxic mutant HTT protein fragment, which contains the expanded polyglutamine stretch [53,54]. This incomplete splicing of mutant HTT from exon 1 to exon 2 can be detected in vivo in diverse HD mouse models [54,55]. Importantly, the level of mis-spliced transcript correlates with the onset of behavioral phenotypes and the striatal disposition of polyglutamine–protein aggregates [55]. Furthermore, in an HD patient’s tissue, this mis-spliced HTT transcript can be detected. Tissues that express this mis-spliced transcript include fibroblasts, cortex, hippocampus and cerebellum [54,56]. Mechanistically, the production of the mis-spliced HTT transcript may be caused by one or more splicing factors that aberrantly bind to the mutant transcript. For example, the splicing factor SRSF6 binds to the mutant transcript [52,54] and manipulation of the expression level of SRSF6 modulates this aberrant splicing event [57]. However, in mouse models, incomplete splicing remained unaffected after the reduction of SRSF6 to 50% of wild-type levels [58]. Thus, it is likely that more than one splicing factor is involved in the mis-splicing of HTT transcripts with expanded CAG repeats.

The second group of aberrant splicing events that occur upon the expression of mutant HTT affects other transcripts; several examples of this group were found in different studies. For example, the *CREB1* transcript is mis-spliced upon the expression of mutant HTT. Mechanistically, *CREB1* mis-splicing involves recruitment of the splicing factor PRPF8 to mutant HTT RNA. Importantly, *CREB1* mis-splicing is also seen in HD brains [52]. Other examples of transcripts that are mis-spliced upon the expression of CAG-repeat constructs include *INSR, SERCA1, CLCN1* and *LDB3*. Here, the overexpression of the splicing regulator MBNL1 partially reverses splicing abnormalities, suggesting that MBNL1 is one of the splice factors that are mechanistically involved here [59]. In a more general approach to identify mis-spliced transcripts in the motor cortex from seven human HD brains via deep RNA-seq analysis, 593 differential alternative splicing events between HD and control brains were detected [60]. In the same study, Lin et al. also evaluated the expression levels of splicing factors in HD patients’ brains. They reported significantly altered expression levels and found that the splicing factor PTBP1 impacts disease-associated splicing [60]. Additionally, in a recent study, Elorza et al. performed intersect-RNA-seq analyses of human postmortem striatal tissue and of an early symptomatic mouse model in which neuronal loss and gliosis were not yet present. This, in combination with a human–mouse parallel motif scan analysis, allowed for the identification of a shared mis-splicing signature that was triggered by the CAG-repeat mutation in both species and gives rise to a total of 949 one-to-one orthologs that are differentially spliced in both human and mouse [61]. In addition, via human–mouse parallel complementary motif searches on common mis-spliced events, the authors concluded a network of candidate upstream splicing factors with reduced protein levels in both species that may be mechanistically involved in mis-splicing [61]. Interestingly, some of these splicing factors, for example, U2AF2 and HNRNPC, were previously found to aberrantly bind to CAG-repeat RNA [52].

All these data indicate that the sequestration of splicing factors to mutant CAG-repeat RNA affects splicing of both the CAG-repeat RNA itself and other transcripts and, thus, one major mechanism of RNA-mediated toxicity is deregulated splicing.

## 5. Aberrant Translation

Upon splicing and maturation of the mRNA, protein translation can be initiated, leading to protein synthesis from the mature mRNA transcript. The translation of expanded CAG-repeat mRNA results in a neurotoxic polyglutamine protein. This event can be seen as a pathogenic function, in connection with the neurotoxicity of the mutant CAG-repeat RNA. 

In addition to splicing factors, translation factors also bind length-dependently to CAG-repeat mRNA such that mutant, i.e., expanded repeats, recruit significantly more protein. One example of protein complexes that aberrantly attach to the mutant CAG-repeat hairpin is a protein complex consisting of MID1 (midline 1), the 40S ribosomal S6 kinase (S6K) and the catalytic subunit of protein phosphatase 2A (PP2Ac) [62]. PP2A is one of the central serine/threonine phosphatases and is therefore responsible for the dephosphorylation of a large amount of the serine/threonine phosphorylated molecules in cells. This phosphatase consists of various subunits. The core is the already mentioned C-subunit (PP2Ac), which dephosphorylates a large number of substrates in vitro. In cells, PP2Ac is bound to either one or two of its regulatory subunits that modify its enzymatic activity and substrate specificity. In this context, MID1 binds to the alpha4 protein, which is one of the regulatory subunits of PP2A. Upon binding, MID1 catalyzes the polyubiquitination of PP2Ac, which marks PP2Ac for degradation in the proteasome. Thus, MID1 acts as a negative regulator of PP2A [63,64].

**Figure 4:**
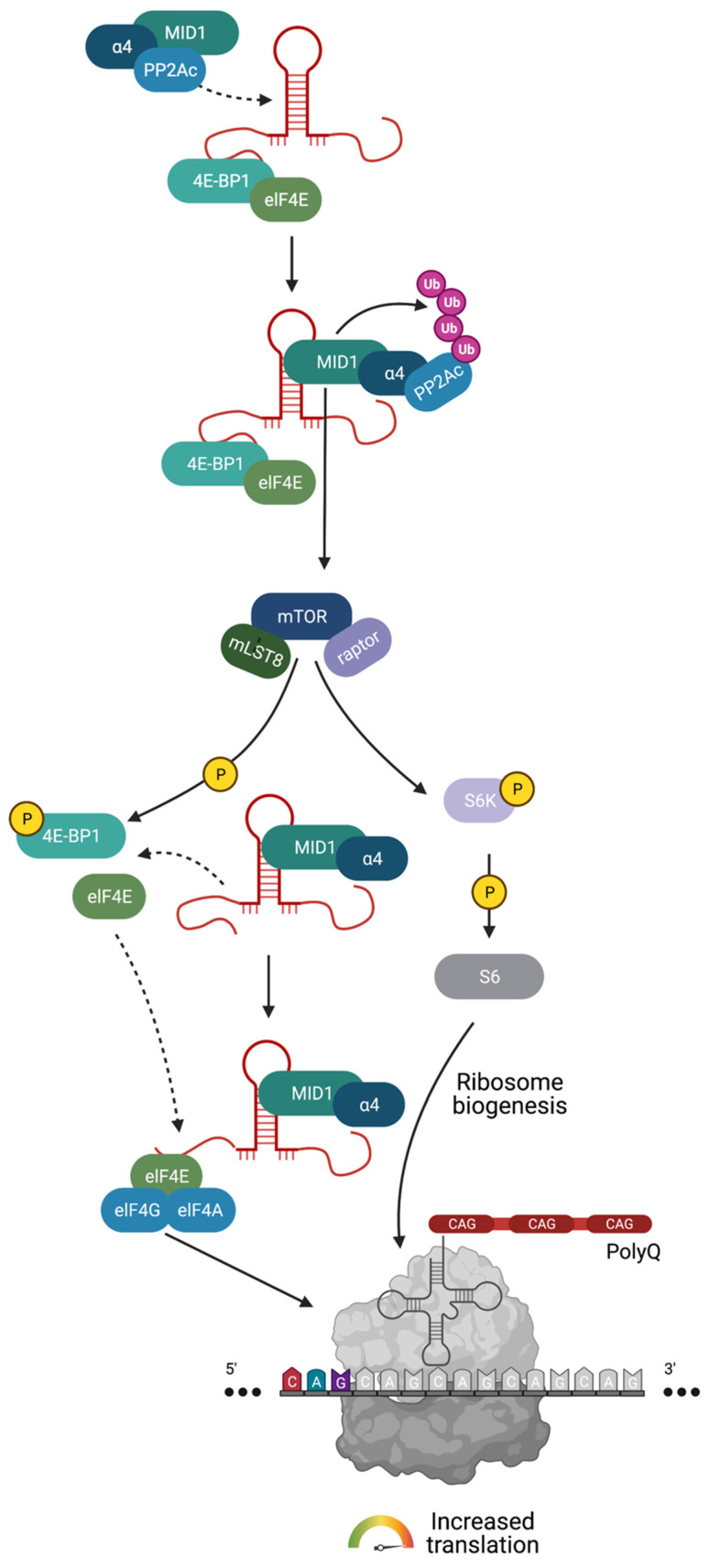
The MID1 protein complex induces translation of HTT mRNA with expanded CAG repeats. MID1 (depicted in teal) attaches to HTT mRNA with expanded CAG repeats (hairpin depicted in red) and mediates the binding of translational regulators, including PP2A (depicted in blue). PP2A and its opposing kinase mTOR (depicted in dark blue) control the phospho-dependent activity of S6K (depicted in lilac) and 4E-BP1 (depicted in light green). 4E-BP1 is a negative regulator of translation that suppresses translation when bound to the 5′ end of an RNA. Its phosphorylation by mTOR leads to the detachment from RNA and the release of this translational block. At the same time, S6K gets activated by phosphorylation via mTOR. Phospho-activated S6K phosphorylates its target S6, which is a subunit of the ribosome. These two mTOR-dependent phosphorylation events promote ribosome assembly on the RNA and thus promote translation. Besides recruiting PP2A and S6K to the RNA hairpin, MID1 induces mTOR activity and simultaneously inhibits the activity of PP2A by inducing its proteasomal degradation. Thus, MID1 indirectly stimulates translation. Created with BioRender.com.

Additionally, MID1 also influences the activity of the kinase mTOR (mammalian target of rapamycin) in the mTORC1 protein complex, which consists of the proteins mTOR, mLST8 (mammalian lethal with SEC13 protein 8) and raptor (regulatory-associated protein of mTOR). Here, MID1 regulates the composition of the mTOR/raptor complex. MID1-deficient cells have an increased level of PP2Ac, which, in the complex with its regulatory B-alpha subunit, inhibits the mTOR/raptor binding and thus the activity of the mTOR/raptor complex. Both proteins, PP2A and mTOR, play an important role in translational regulation [65]. Among other functions, mTOR phosphorylates and activates the protein S6K, which plays an important role in the induction of translation [66]. Activated S6K phosphorylates and activates its target protein S6, a ribosomal subunit. At the same time, the negative translation regulator 4E-BP1 (eukaryotic translation initiation factor 4E (eIF4E)-binding protein 1) is inactivated by phosphorylation via mTOR. As a result, 4E-BP1 is released from the 5′ end of the RNA and loses its attachment to its interaction partner elF4E (eukaryotic translation initiation factor 4E). elF4E can then bind to elF4G and elF4A and the resulting complex binds to cofactors and to the 5’UTR. Consequently, structural changes affect the conformation of the RNA in conjunction with the translation initiation factor complex such that the ribosomal proteins are recruited, the ribosome is assembled and translation is enabled. mTOR and PP2A, which are regulated by MID1, can direct the translation of MID1-bound mRNAs by changing the phosphorylation status of S6K and 4E-BP1. Thus, the repeat-length-dependent binding of the MID1 protein complex leads to an increased translation rate of the mutant CAG-repeat mRNA. Therefore, the expanded CAG-repeat mRNA can recruit translation factors and stimulate its own translation rate, which can be seen as a toxic gain of function of the mutant CAG transcript (Figure 4). Interestingly, MID1 binds to multiple CAG-repeat mRNAs regardless of the repeat-flanking sequences so that translation induction by MID1 occurs in cell models of multiple CAG-repeat diseases, such as HD and spinocerebellar ataxia types 2 (SCA2), 3 (SCA3) and 7 (SCA7) [67]. Consequently, inhibiting the binding of the MID1 complex could be an encouraging mechanism for suppressing the increased translation of expanded CAG-repeat mRNA in several CAG-repeat diseases, including HD.

Generally, the translation of mRNA is initiated at the start codon (AUG). This also applies to the open reading frame of the RNAs with expanded CAG repeats, which are translated into polyglutamine protein. Until 2011, it was assumed that, in the frame with the start codon, only the polyglutamine protein is produced from the mutant CAG repeats. However, in 2011, Zu et al. showed an AUG-independent possibility of translation since mutation of the only AUG codon in ATXN8 transcripts with CAG-repeat expansion did not prevent protein translation [68]. The repeat-associated non-AUG (RAN) translation occurs in GC-rich RNA sections. Such GC-rich regions are not only typical for CAG-repeat diseases, such as HD, but also occur in diseases caused by expansion of other repeats (such as CXG or GGGGCC).

**Figure 5:**
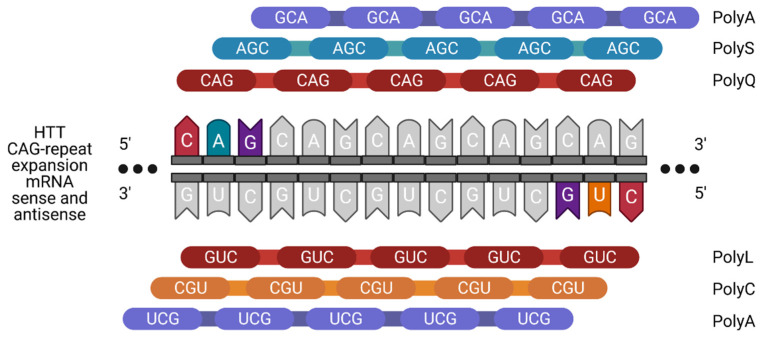
Schematic overview of an RAN translation. Mutant HTT is transcribed from both sense and antisense strands and both resulting transcripts (CAG and CUG repeats) fold into hairpin structures that aberrantly recruit RNA-binding proteins (RBPs). This leads to an RAN translation (repeat-associated non-AUG translation), which is a special form of translation that does not require an AUG start codon but can start at any base in all 3 reading frames. This leads to translation of aberrant protein species in all 6 reading frames: polyalanine (polyA), polyserine (polyS), polyglutamine (polyQ), polyleucine (polyL) and polycysteine (polyC) proteins. Created with BioRender.com.

Since both the sense and the antisense strands of the DNA can be transcribed into RNA, bidirectional GC-rich mRNAs can be formed from the same gene locus. Since no AUG start codon is required for the RAN translation, these two complementary mRNAs can theoretically be translated. Thus, two expanded repeat mRNAs (sense and antisense) can be translated in all six reading frames into six different potentially toxic proteins (Figure 5).

Four mutant HTT-RAN translation proteins (polyalanine, polyserine, polyleucine and polycysteine) from the sense and antisense transcripts were found in 2015 by analyzing human HD autopsy brain samples [69]. This finding was followed by several research projects that led to inconsistent results. For example, one study was conducted on C. elegans expressing HD-RAN homopolymers. These contained codon-varied tags and different CAG-repeat lengths. This study showed that only the polyL protein induced significant toxicity depending on the CAG-repeat length, although all RAN translation proteins aggregated [70]. In contrast, different results were observed in the brains of two different mouse models: (1) a model that enabled RAN translation but did not express HTT with the polyglutamine tract translated from the start codon, and (2) a model that expressed N-terminal polyglutamine HTT. Interestingly, no RAN translation products could be identified and only those mice that expressed N-terminal polyglutamine HTT showed an HD phenotype [71]. It should be noted that these studies are difficult to compare due to their different model systems and experimental set-ups. Further experiments are needed to conclude the relationship between RAN translation and neurotoxicity in HD and other neurodegenerative CAG-repeat disorders.

Taken together, all the above-mentioned experiments suggest that mutant CAG-repeat mRNAs can aberrantly recruit translation factors and promote their own translation, not only in the open reading frame defined by the AUG start codon but also in different reading frames starting at the repeat sequence.

Besides this aberrant translation triggered by the CAG-repeat mRNA, translation in HD is also affected by two further pathogenic mechanisms that are linked to the mutant HTT protein. On one hand, mutant HTT protein interacts with ribosomal proteins and can lead to ribosomal stalling, thereby disrupting translation [72]. On the other hand, mutant HTT protein represses the expression of the mitochondrial metabolic regulator peroxisome proliferator-activated receptor gamma co-activator 1α (PGC-1α) [73,74]. In addition to its metabolic functions, PGC-1α is involved in ribosome biogenesis. Consequently, in HD, ribosome biogenesis and thus protein synthesis is reduced, as shown, for example, in the muscle biopsies of HD patients [75]. In summary, the general translation machinery is impacted by both mutant HTT mRNA and mutant HTT protein, with the mutant HTT mRNA pushing towards its own translation and the mutant HTT protein impacting ribosomes. Thus, the general protein synthesis is disturbed by two hits in HD.

## 6. Deregulation of the microRNA Machinery

**Figure 6:**
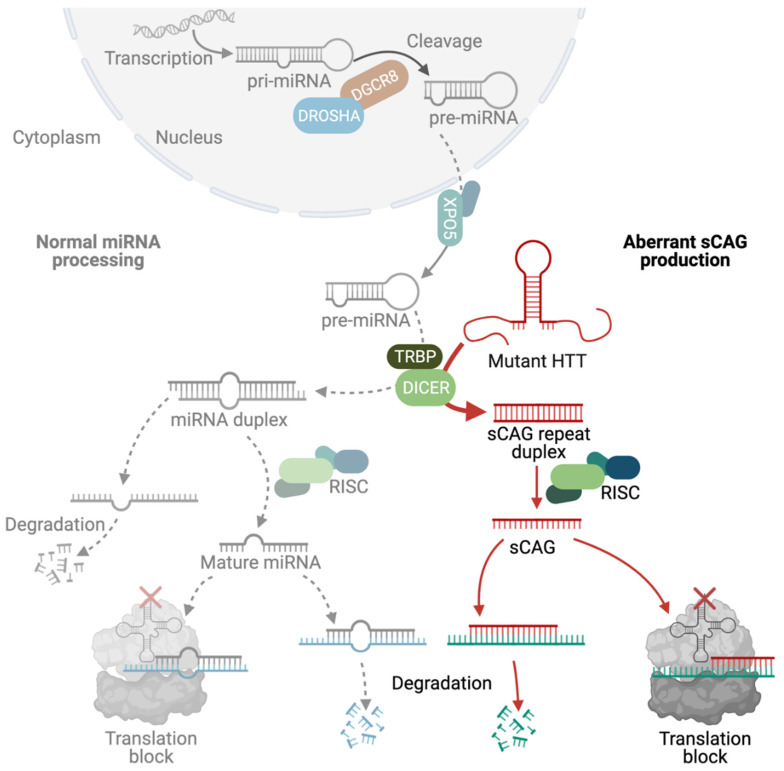
Deregulation of the miRNA machinery and production of sCAGs. miRNAs bind to complementary sequences in their target mRNAs of protein-coding genes. miRNAs are transcribed as primary RNA (pri-miRNA) that undergo serial cleavage steps within the nucleus due to enzymes including DROSHA and DGCR8, as well as outside the nucleus due to enzymes including DICER and TRBP. This cleavage results in small double-stranded RNAs with 18–23 nucleotides, of which the mature miRNA associates with the RISC complex, while the antisense strand is degraded. The miRNA then guides the RISC to its complementary target mRNA, where it either induces degradation of the target mRNA or suppresses its translation. The mutant HTT RNA hairpin aberrantly recruits DICER, which leads to the cleavage of the long double-stranded CAG repeats into fragments with 22 nt, termed sCAGs. These sCAGs associate with the RISC and recruit the RISC to mRNAs with complementary sequences. This, in turn, results in the aberrant translational inhibition of the mRNAs with CUG-motifs. Created with BioRender.com.

Another group of RNA-binding proteins that get trapped by mutant CAG-repeat RNA, resulting in aberrant function, includes RNA processing enzymes that are involved in the generation of small non-coding RNAs. Interestingly, more than 90% of the human genome is transcribed but does not translate into protein [76]. These transcripts are called non-coding RNAs and, beyond the fact that it is still not clear whether all of these transcripts are functional, there are multiple examples of molecular functions of non-coding RNAs [77]. For example, microRNAs (miRNAs) are endogenously expressed short non-coding RNAs that downregulate gene expression of protein-coding genes in several cellular processes [78,79,80]. miRNAs bind to complementary sequences in their target mRNAs of protein-coding genes. miRNAs are transcribed as full-length transcripts called the primary RNA (pri-miRNA) that go through a sequence of cleavage steps to produce small double-stranded RNAs of 18–23 nucleotides. The first intranuclear cleavage step involves a protein complex containing the Drosha and DGCR8 (DiGeorge syndrome critical region in gene 8) and results in an RNA fragment of 60–70 bp, which is referred to as precursor miRNA (pre-miRNA) [81,82,83]. The pre-miRNA is then transported to the cytoplasm, passing the nuclear pore with the help of exportin 5 [84,85]. The second cleavage step is carried out by the enzyme DICER in combination with TRBP (trans-activation response RNA-binding protein) and results in an 18–23 bp RNA duplex of the mature miRNA and its antisense strand. While the antisense strand is released and degraded [86,87,88,89], the mature miRNA then associates with and guides the RNA-induced silencing complex (RISC) to its complementary target mRNA. The miRNA–RISC complex can either induce degradation of the target mRNA or suppress its translation [90,91,92,93,94] (Figure 6).

Interestingly, some of the enzymes involved in miRNA cleavage aberrantly bind to mutant HTT RNA at its CAG-repeat region, resulting in a DICER-dependent production of small CAG-repeated RNAs (sCAGs) with <21 nucleotides. Of note, both the CAG-repeat RNA hairpin structure and the double-stranded RNA resulting from bi-directional transcription in the sense and anti-sense directions are appropriate substrates for DICER [95,96]. Reporter studies suggest that sCAGs functionally lead to loading of the CAG-nucleotide to the RISC [27] and, finally, to silencing of CUG-containing RNAs [97]. However, general evaluation of HD brain transcriptome profiles has not revealed enrichment for CUG-containing genes [98]. Nevertheless, the cellular expression of sCAGs is sufficient to induce neuronal DNA damage by misregulating the expression of NUDT16 and the application of a compound that blocks duplex CAG RNA rescues both NUDT16 expression and DNA damage in HD mice [99]. Similarly, the blocking of sCAGs by anti-miRs also reduces toxic effects, supporting a key role of sCAGs in HTT-RNA-mediated toxicity [97]. Thus, their involvement in pathogenesis is very much anticipated. Strikingly, increased levels of sCAGs are also found in HD patient brain tissue [97]. The detrimental effect of sCAGs can be explained in two ways: either by the silencing of CTG-containing genes or by aberrant recruitment of the miRNA-cleavage machinery and, thus, deregulation of miRNA synthesis. Indeed, the expression of several miRNAs is deregulated in HD [100], as well as other CAG-repeat-expansion diseases [101,102,103]. However, the exact molecular contribution of the miRNA machinery and sCAGs in pathogenesis requires further study, especially since another layer of complexity is observed in HD, where mutant polyglutamine protein also interacts with a member of the RISC complex, namely, Argonaute (Ago2) [104].

Another layer at which the miRNA machinery can affect cellular disease-mechanisms is by changing the protein expression of RNA-binding proteins that aberrantly bind to mutant HTT RNA and exhibit abnormal function in association with the CAG-repeat RNA. For example, miRNAs that regulate the expression of the MID1 complex [105] could change the translation rate of mutant HTT by repressing MID1 and, thus, could counteract the increased translation of polyglutamine protein. Another example involves miRNAs targeting important modifiers in HD pathogenesis, such as BDNF [106]. Indeed, differential expression of miRNAs was detected in HD tissue [107]. Future studies should aim at identifying the exact mechanisms of miRNA-dependent processes in disease development.

## 7. Mitochondrial RNA

While in all the above-mentioned studies, the aberrant interaction of mutant HTT mRNA was described, abnormal RNA-function in HD also occurs at a different level, namely, in the mitochondrial RNA (mtRNA). Generally, mitochondria are important for cell viability and several lines of evidence have connected mutant HTT protein with mitochondrial dysregulation [108,109]. For example, striatal cells expressing mutant HTT protein exhibit a substantial increase in the mitochondria-released reactive oxygen species (ROS). As mtDNA is a major target of oxidative stress in HD, a dramatic decrease in the mtDNA in mutant HTT cells is seen [110]. Additionally, defects in mitochondrial Ca^2+^ handling contribute to HD pathogenesis [111]. Interestingly, the suppression of p53 leads to a substantial reversal of mutant HTT-induced mitochondrial depolarization and cytotoxicity [112], indicating an integral role of p53 in mitochondrial dysfunction caused by mutant HTT protein. While these effects on mitochondrial function are linked to the aberrant function of the mutant HTT protein, in this review, we aimed to focus on RNA-mediated aberrant mechanisms. In this respect, there is evidence that mitochondrial RNA (mtRNA) plays an important role in imparting mitochondrial dysfunction. In an interesting experiment conducted by Lee et al. cell-type-specific transcriptomic analyses were done in order to study the gene expression changes in human HD and mouse models of HD by single nuclear RNA sequencing (snRNA-seq) and translating ribosome affinity purification (TRAP). They conducted striatal cell-type-specific transcriptomic studies in both human and mouse models. In HD, they observed the cytosolic release of mtRNA, a potent innate immunogen in striatal spiny projection neurons that led to the upregulation of innate signaling responses. They also showed that the released mtRNAs bind to innate immune sensor protein kinase R (PKR), which, when inhibited, could lead to the suppression of innate immune pathway signaling [113]. Recently, a group of scientists developed a new human neuronal cell model by using neural stem cells (ReNcell VM NSCs). These cells were transduced to express HTT exon 1 with variable lengths of CAG repeats. Investigations showed repeat-length-dependent formation of intranuclear inclusions during neurogenesis, as well as marked mitochondrial dysfunction [114]. Taken together, these observations suggest that not only the mutant CAG-repeat mRNA transcript plays a role in RNA-mediated toxicity but mtRNA is also deregulated and pushes pathogenic mechanisms. However, further studies that focus on elucidating the mechanisms of mtRNA-mediated neurotoxicity are required to understand the mtRNA’s toxic gain of function in HD.

## 8. Compounds

Given the fact that in HD, at least two neurotoxic gene products (the mutant transcript and the mutant protein that is cleaved into two neurotoxic N- and C-terminal fragments) contribute to neurodegeneration, an ideal therapeutic approach would target both the mutant RNA and protein. A two-photon Ca^2+^ imaging study in Hdh150 knock-in mice by Arnoux et al. showed neuronal hyperactivity in the visual cortex well before disease onset (VFDO), suggesting that therapeutic intervention may be required at an early stage. Small bioavailable molecules that are capable of crossing the blood–brain barrier would be ideal therapeutics to inhibit such pathogenic cellular processes, for example, by reducing aberrant recruitment of RBPs to mutant HTT RNA. One compound that was studied in the context of VFDO is dimethylbiguanide metformin, which is an FDA-approved, low-cost type II diabetes drug. Metformin suppresses mutant HTT translation via interfering with the MID1 complex. This occurs through the metformin-mediated disassembly of the MID1 complex that leads to the activation of PP2A. This, in turn, decreases protein translation and, thus, the protein level of mutant HTT both in cell cultures and in vivo. In addition, metformin treatment reverses early-onset network dysregulation in HD mice [115]. In 2017, metformin was also shown to improve central phenotypic traits by normalizing ERK signaling in a mouse model for a CGG-repeat disorder, namely, fragile X syndrome [116]. Thus, metformin seems to be promising for the therapy of CXG repeat expansion diseases, including HD. However, to assess metformin‘s effect in HD patients, clinical studies are required and, thus, further research is needed to show the consequences of its long-term administration in HD patients.

Another molecule that was studied in HD models is furamidine. In silico identification of CAG binding ligands led to the identification of furamidine, which binds to HTT exon1 RNA in vitro. The RNA binding of one known mutant HTT interactor, namely, MID1, was blocked by furamidine, as shown, for example, in in vitro RNA protein pulldown experiments. As a consequence of blocking MID1 binding to mutant HTT RNA, the compound also reduces HTT translation and protein levels in an HD cell-line model. However, furamidine binding is not specific for CAG-repeat RNA. In addition to CAG repeats, it also binds AU-RNAs, CUG-RNAs and the DNA minor groove [117]. Thus, undesirable side effects are expected, which provide an argument against HD treatment with furamidine.

Another interesting compound for HD therapy is one of the flavonoids. Flavonoids are known for their health benefits and can act as antioxidants, anticancer agents, neuronal protection and neurodegenerative disease prevention. One flavonoid, namely, myricetin, was shown to interact with the CAG motif and, thus, prevent both the translation of mutant HTT protein and the sequestration of MBNL1. This happens through the interaction of myricetin with RNA via base stacking at the A–A mismatch of the hairpin structure from expanded CAG repeats. Additionally, in a 3-nitropropionic acid (3-NP)-induced HD rat model, the oral supplementation of myricetin prevented mitochondrial dysfunction and oxidative stress, along with motor deficits [118]. Further research will show whether the therapeutic results on myricetin can be translated from rats to humans.

In 2019, Khan et al. designed and synthesized several pyridocoumarin derivatives to attach to CAG motifs. Two of these derivatives turned out to be promising: both molecules showed higher affinity and selectivity for expanded CAG-repeat RNA when compared to regular duplex AU-paired RNA. Interestingly, both molecules are cell-permeable and exhibit low toxicity to healthy fibroblasts. Additionally, they are also capable of reducing the level of polyglutamine aggregation in cell cultures. Further research will show whether these compounds have the same beneficial effects in vivo.

## 9. Antisense Oligonucleotides

Antisense oligonucleotides (AONs) are short (8–50 bases), synthetically produced nucleic acids that bind complimentarily through Watson–Crick base pairing to a specific sequence within their target RNA. Depending on their chemical design and target sequence, AONs act through different mechanisms, including mechanisms that promote degradation of the target mRNA, mechanisms that lead to translational inhibition or mechanisms affecting splicing. For example, if a DNA AON binds its target mRNA, thereby forming an RNA–DNA heteroduplex, this duplex is detected by endogenous enzymes, such as ribonuclease H (RNase H), which cleaves the RNA strand. If an AON binds to its target pre-mRNA at intron/exon junctions, it can mask splicing enhancers and repressor sequences, thereby modulating splicing events. If an AON binds to its target mRNA, it can also sterically block the 40S and 60S ribosomal subunits from attaching, thereby blocking translation (reviewed in [119]). AONs have been in clinical use for many years. For example, in 1998, formivirsen obtained approval for use in the treatment of cytomegalovirus retinitis in patients with immunodeficiency [120].

Although a lot of research has been conducted and several attempts have been made to develop therapeutic approaches, no disease-modifying treatment options are routinely available to date, and current medical management is restricted to supportive care. This may change with the development of HTT-lowering AONs. Several reports performed in mouse models showed that AONs can be beneficial.

For example, Kordasiewicz et al. showed that the injection of an AON into the cerebrospinal fluid of a symptomatic HD mouse model delayed disease progression [121]. The AON in this study not only reduced the expression of mutant HTT but also that of the normal HTT allele. In a different approach, Ostergaard et al. developed an AONs treatment approach that is specific for the mutant HTT allele by targeting single nucleotide polymorphisms (SNPs) that are associated with the repeat expansion [122]. Another way to specifically target the disease allele is to target the CAG-repeat region. Indeed, using a (CUG)_7_ AON showed beneficial effects in HD models, along with models of other CAG-repeat-expansion diseases, such as spinocerebellar ataxia type 1 (SCA1) and SCA3 [123,124,125]. Based on these encouraging results, AONs represent an interesting HTT-lowering strategy for HD. If AONs are used to target the CAG repeat, it could not only prove to be a promising therapeutic tool to diminish the mutant HTT in HD but also help in perturbing the effects of other polyglutamine diseases.

## 10. Clinical Trials and Novel Therapies

To this point, there is no therapy that can cure CAG-repeat diseases, such as HD. The currently available treatments impart only symptomatic relief but do not stop disease progression. Through physiotherapy or speech therapy, the patient learns to manage involuntary movements or speech difficulties. Neuroleptics or tetrabenazine (indirectly acting as antidopaminergic) are used as medication. These substances help to inhibit uncontrollable muscle movements [10]. Psychotherapies are used to treat depression. In addition, genetic counseling can be of importance for the families of the patients.

Various therapeutic approaches for curing HD are being explored in current research projects and clinical studies. A primary goal is to develop therapies that will take effect before symptoms appear, as it is not certain that the symptom-free phenotype can be restored later in HD. During this period, the post-transcriptional level offers opportunities for promising therapeutic approaches since the targeting of the mutant transcript and, consequently, the lack of the mutant protein would deprive most of the known pathomechanisms.

One option for targeting the mutant transcript involves AONs. There are two different approaches using AONs that are currently used in clinical trials for HD treatment. In the first approach, AONs targeting the HTT transcripts from both alleles (normal and mutant) are used. An example is an AON called tominersen. In preclinical studies on model organisms, the use of tominersen led to significant changes. After 14 days of treatment of BACHD mice (which express human mutant HTT) with the AON tominersen, the HTT mRNA level was reduced by up to 80%. This reduction lasted for 16 weeks. In addition, symptomatic improvement was observed in three different HD mouse models. In this context, the motor deficits in young animals were almost completely restored. Next, this AON was tested in clinical trials. In a phase 1b/2a clinical study conducted in 2015, the tominersen doses were administered intrathecally. Notably, this Ionis-Pharmaceuticals-funded study showed that tominersen decreased levels of the mutant HTT protein in cerebrospinal fluid (CSF) in patients with early stages of HD. Thus, this attempt awakens new hope for a curative treatment. However, it is unclear whether lowering wild-type HTT in an organism has long-term consequences. The phase 3 study on tominersen (GENERATION-HD; NCT03761849; sponsored by Hoffmann-La Roche), which aimed to clarify these consequences, began in January 2019. This trial was planned to end in 2022 but was terminated in March 2021 due to poor tolerance of the maximum dose [126]. Since the AON used in this trial targets both mutant and normal alleles and HTT is an essential protein, it may be that reducing the HTT protein below a critical level cannot be tolerated over a prolonged period of time. Thus, it may be helpful to target the mutant allele only while keeping the normal allele untouched. 

One allele-specific approach to target only the mutant allele is to design AONs directed against heterozygous SNPs on the disease allele. Many SNPs have already been found in the HTT DNA sequence. With the help of special sequencing techniques, it can be determined which allele carries which SNP. This enables the generation of allele-selective AONs that target only the mutant allele HTT. It is estimated that AONs that bind to the three most common SNPs of mutant HTT would be sufficient to treat approximately 80% of the populations of European ancestors, but the exact AON must be selected specifically for each patient. In addition, the similarity of the overall sequences (mutant HTT versus normal HTT) limits the selectivity of the AONs, which means that the allele specificity of the AONs may not be 100%. However, the general concept of targeting the disease allele and maintaining a functionally normal HTT is a promising approach that was tested in phase 1 and phase 2 clinical trials (PRECISION-HD2; WVE-120102; NCT03225846) until spring 2021. The therapy was generally well tolerated, with most adverse events being mild or moderate in intensity (for example, headache, injection-associated pain, back pain and dizziness) and occurring mainly in the group receiving the highest dose. However, due to the lack of consistent and significant reductions in mutant HTT, the development of WVE-120102 was stopped. As an alternative approach, Wave life science is planning a clinical phase 1b/2a study that is supposed to start in 2021 to test WVE-003. WVE-003 is a next-generation AON that targets a specific single nucleotide polymorphism (SNP3) that is commonly found on the expanded CAG allele (approximately 40% of HD patients carry SNP3 in association with the CAG expansion mutation). This AON has different phosphate modifications in the backbone to improve its function (e.g., increase potency, exposure and durability) [127].

Like AONs, RNAi are nucleotide-based molecules that bind to mRNAs and cause the cell to degrade the bound transcript. RNAi uses RNA-based therapeutic molecules: short interfering RNA (siRNA), short hairpin RNA (shRNA) or microRNA (miRNA). RNAi compounds are also under investigation in the area of HD treatment. It should be noted that double-stranded siRNA has limited distribution and cellular uptake in the CNS compared to single-stranded AONs. Viral vectors, such as the adeno-associated virus (AAV), offer an advantage regarding delivering the siRNA or miRNA into the brain parenchyma through stereotactic surgery [128]. This type of administration is difficult but can be considered a one-time treatment because virally administered RNA therapeutics stably transduce CNS cells and these produce the RNAi molecules that suppress mutant HTT. Such medication is considered a form of gene therapy since the siRNA/miRNA coding sequences integrate into the genome. The reduction of HTT via AAV-supplied siRNA or miRNA is effective in HD rodent models [129], as well as in human and primate (wild-type rhesus monkeys) studies. Of note, after extensive histological, biochemical and clinical tests in primates, no significant side effects were found, other than the risk associated with any neurosurgical administration technique [130]. However, the limited tissue distribution is a significant challenge for RNA-based therapy that requires consideration of multiple injection sites or the prioritization of one brain region. There are currently no clinical studies of RNAi on the reduction of mutant HTT RNA in humans.

## 11. Concluding Remarks and Future Directions

**Figure 7:**
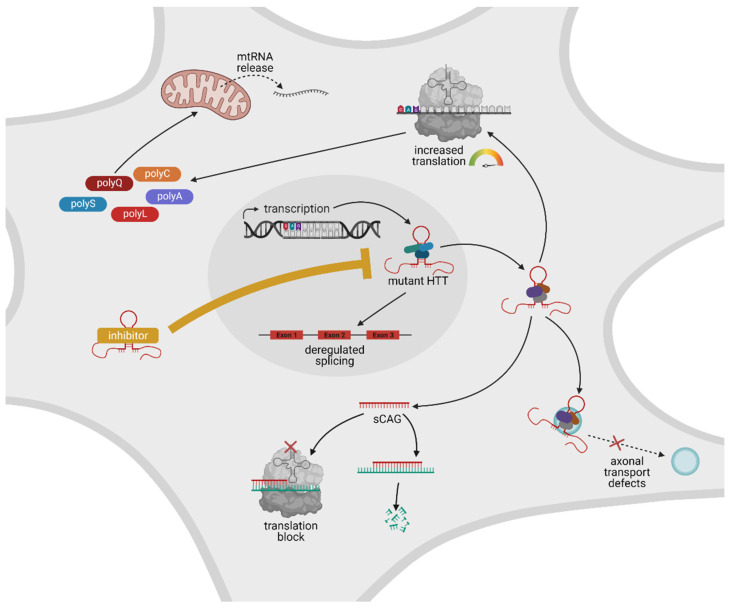
Schematic overview of RNA-mediated pathogenic mechanisms and RNA-targeting approaches. Within the nucleus, mutant HTT is transcribed. The respective CAG-repeat-containing transcript folds into an aberrant hairpin structure (depicted in red) that can trap diverse RNA-binding proteins, both within the nucleus and the cytosol. This leads to a variety of aberrant mechanisms: recruitment of splicing factors within the nucleus leads to deregulated splicing; recruitment of translation factors in the cytosol results in increased translation of polyQ protein, as well as polyA-, polyS-, polyL- and polyC peptides; recruitment of proteins from the miRNAs machinery leads to the generation of small CAGs (sCAGs), which can block translation of CUG-containing transcripts; binding to proteins that are involved in the formation of RNA granules leads deregulated axonal transport; expression of mutant HTT leads to an aberrant release of mitochondrial RNA (mtRNA). Novel treatment strategies use RNA-targeting drugs that can counteract these aberrant RNA–protein interactions. The advantage of RNA-targeting therapeutic strategies (visualized as the inhibitor depicted in yellow) is that both the above-mentioned RNA-dependent disease mechanisms and the production of aberrant protein species are blocked simultaneously. Created with BioRender.com.

Taken together, HD is an incurable autosomal-dominant inherited condition caused by a CAG-repeat-expansion mutation that encodes two neurotoxic gene products: an expanded CAG-repeat transcript and a polyglutamine protein that is cleaved into two neurotoxic fragments, namely, an aggregate-prone polyglutamine N-terminal fragment and a non-polyglutamine C-terminal fragment. Of note, both neurotoxic gene products (transcript and protein) are expressed together in disease cells; as such, they will most likely also influence each other’s production: the mutant transcript obviously encodes the mutant protein and pushes its own translation, but conversely, the protein can also influence the transcript. For example, the C-terminal cleavage fragment of mutant HTT binds and inactivates dynamin 1 at the ER, thereby causing ER dilation and toxicity [20]. ER stress, on the other hand, is associated with differences in RNA processing, such as unconventional splicing or differential processing of small RNAs [131,132]. Thus, it is tempting to speculate that mutant HTT protein may also be involved in the dysregulation of its own transcript. However, since both gene products contribute to a variety of toxic gain of function mechanisms, an ideal therapy should target both the mutant transcript and protein (Figure 7).

Recent pre-clinical research and clinical trials show that RNA-targeting drugs have great potential with regard to HD therapy. One future goal of RNA-targeting drugs would be to overcome the administration via injection into the CNS; alternatively, a bioavailable molecule that could be used for example for oral administration. However, it may take several more years before such compounds can be used in routine therapies.

Additionally, further studies are required to assess the long-term side effects and their consequences. Since HTT is an essential protein and its knockout is embryonically lethal, the long-term suppression of the HTT gene products by RNA-targeting drugs, which may be necessary over decades when patients are chronically treated, may come with unwanted side effects. Recent clinical trials targeting both normal and mutant alleles have given the first evidence that lowering HTT below a critical level may not be tolerated over a prolonged period. Thus, allele-specific targeting may be a better option. However, long-term clinical observations using such allele-specific targeting drugs are required. Similarly, although HD is a late-manifesting neurodegenerative disorder, recent studies of mutation carriers suggest that the CAG-repeat expansion within the HTT gene affects neurodevelopment. In tissues from both human fetuses and mouse embryos that carried an HTT mutation, abnormalities in the developing cortex, such as mislocalization of mutant HTT protein and other junctional complex proteins; defects in the neuroprogenitor cell polarity; and differentiation, abnormal ciliogenesis, and changes in the cell cycle are detectable. Thus, HD has a neurodevelopmental component as well and is not solely a neurodegenerative disease [133]. Therefore, future therapeutic approaches should include aspects of early diagnosis and treatment, potentially even decades before the first symptoms of neurodegeneration appear.

Finally, several of the pathogenic mechanisms involved in HD are also detectable in other CAG-repeat-expansion disorders. These include the deregulated expression of miRNAs [101,102,103], RAN translation [68], MID1-dependent translation induction [67] and aberrant alternative splicing [28,59,134]. Thus, these aberrant RNA-dependent functions represent a general phenomenon in CAG-repeat diseases and may even be further translated to other CXG-repeat expansion disorders. Consequently, targeting expanded CAG-repeat mRNA represents a promising approach in several CAG-repeat diseases, including HD.

## Data Availability

Not applicable.
